# Construction of web-based remote diagnosis system using virtual slide for routine pathology slides of the rural hospital in Japan

**DOI:** 10.1186/1746-1596-8-S1-S4

**Published:** 2013-09-30

**Authors:** Ichiro Mori, Takashi Ozaki, Yasuteru Muragaki, Takatoshi Ibata, Hiroshi Ueda, Toshihito Shinagawa, Yoshiyuki Osamura

**Affiliations:** 1 Department of Pathology, Mita hospital, International University of Health and Welfare, Minato-ku, Tokyo, Japan; 2 Department of Pathology, Wakayama Medical University, Wakayama, Japan; 3 Shingu Municipal Medical Center, Wakayama, Japan; 4 Kawasaki Municipal Ida Hospital, Kanagawa, Japan

## Background

In recent years, Japan has been short of pathologists. There are about 2,000 board-certified pathologists against 130 million people. Most pathologists are working in the urban area and only few are available in rural regions. Telepathology in Japan developed under these circumstances, mainly for intraoperative frozen section diagnosis where no pathologist is available. We have been performing frozen section telepathology for more than 10 years, and achieved fairly good results [1].

In this work, we constructed a web-based remote diagnosis system using virtual slide targeting routine pathology slides.

## Material and methods

**Figure 1:**
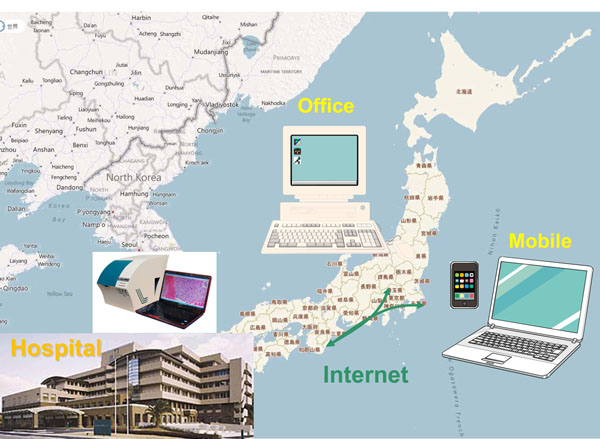
**VS telepathology of routine HE slides.** We made primary diagnosis of VS of routine HE slides of a hospital 500 km away from Tokyo through internet.

The hospital has about 2,000 histology specimens every year, and there are three pathology technicians in the pathology laboratory. Part-time pathologists used to visit the hospital twice a week. We started this project because one of the two pathologists moved, and could no longer go to the hospital. We used LINCE from CLARO (Aomori, Japan), a tiling type VS scanner. To scan slides, we fixed the objective lens to 40x. We limited the target to the biopsy specimens. Pathology technicians scaned paraffin slides and stored VS to the shared folder of their server. They also scanned the clinical application form, and saved the PDF files to the same folder. Personal information is excluded for security reasons. The pathologists opened a shared folder of the server through the internet from Tokyo, 500 km from the hospital (see Figure 1). First, we checked the serial pathology numbers of VS and application form. Then we read the application form, observed VS, making a primary diagnosis report using word processor containing the serial pathology number in the report. The diagnosis reports were then stored to the same shared folder. This was done using remote VPN software. Technicians copied the report and pasted it to the reporting system to complete diagnosis.

In some difficult cases, we discussed with pathologist who visits the hospital once a week, and compared the diagnosis between VS and microscope.

## Results and discussion

We started this system in July 2011, and continue to diagnose about 80 cases each month resulting in more than 800 primary diagnosis. The VS images were clear enough and we could diagnose most of the routine HE slides with no incident. No image degradation happened due to data transfer. Because VS are stored in the server and connected to the web, we can manage our time and location for diagnosis easily. I have also been able to view VS images from Europe using the internet at my hotel.

As a setting of VS scanner, we first tried to use 20x objective lens. But in some delicate cases, the images were frustrating for us. Since we are unable to predict the delicate cases, we fixed objective lens to 40x.

**Figure 2:**
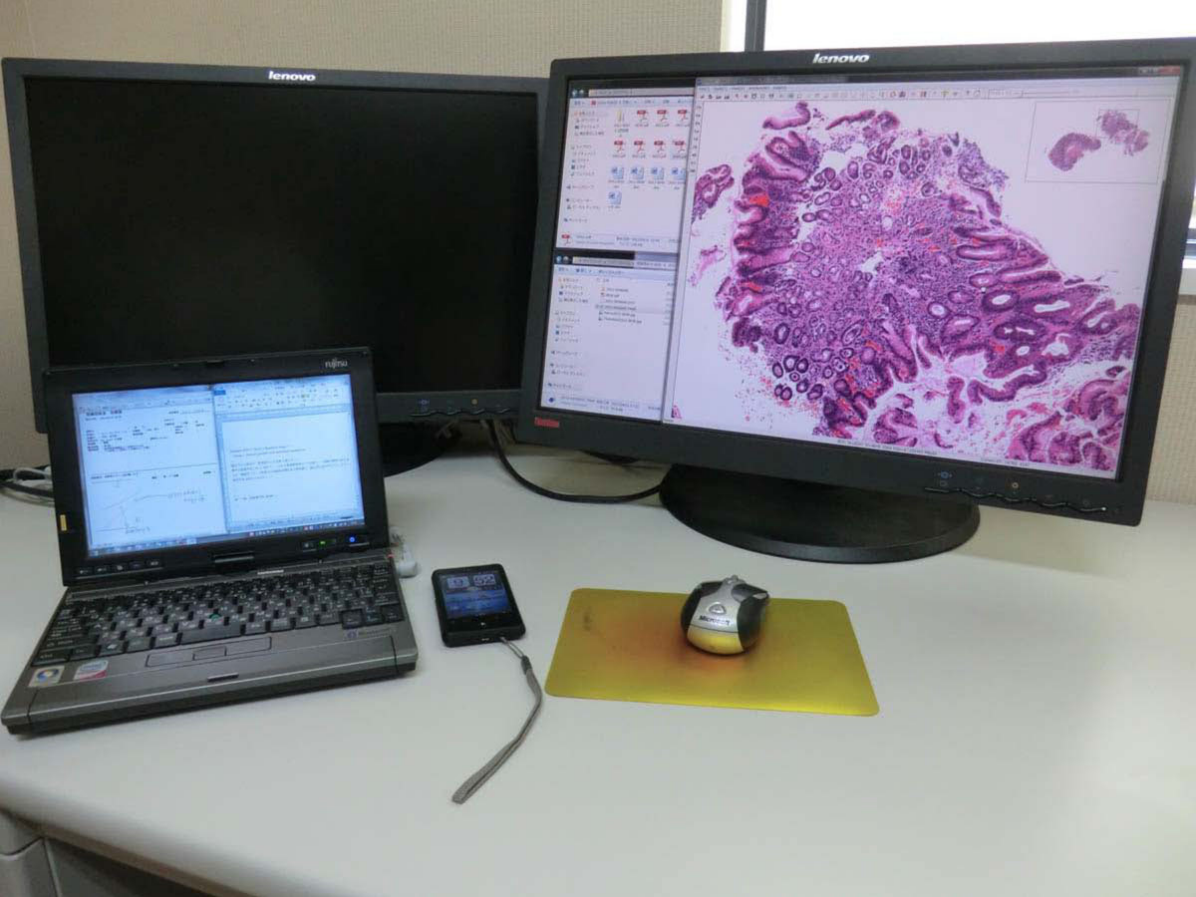
**Actual working desk of remote diagnosis** Notebook PC was connected to the web through mobile telephone network. Another display is also connected to the notebook PC, and diagnosis was done under dual display condition.

When performing a diagnosis on the PC, we usually open 5 windows, 1) clinical application form, 2) VS viewer, 3) word processor window to write diagnosis report, 4) shared VS data folder, and 5) folder to temporary store the diagnosis report. This is too much to display on 10 inch screen notebook PC, or a 22 inch desktop screen. As a result, we are using a dual display system (see Figure 2).

To check the discrepancies between direct microscope and remote VS diagnosis, we looked at some difficult cases and discussed together. The degrees of coincidence were fairly good.

The biggest issue was the data transfer speed. The response of the VS differed each time, sometimes it was really slow whilst at other times it was very fast. When comparing VS observation using broadband of 50-60 Mbps to a mobile telephone network of 1-5 Mbps, the difference was small. We also changed the internet connection of the hospital’s VS server which resolved the issue dramatically. There should be bottleneck somewhere between the VS server and the web this time.

One big trouble was misunderstanding of the slide direction of VS. We put multiple small specimens on one glass slide, and numbered them from the label side. Because our VS viewer does not show thumbnail images with labels, a pathologist cannot find which side in #1 on viewer. Technicians usually set glass slides to the VS scanner with the slide label on the right hand side, which makes the right side specimen #1. One day, a glass slide was accidently placed onto the scanner the opposite way round. There were 4 colon specimens on the VS, and the 2^nd^ specimen from the right showed cancer. The specimen #2 was taken from the ascending colon, but actually it was the specimen #3 taken from the descending colon. Luckily we noticed this before the operation.

According to the Japanese Medical Practitioners Act, primary pathology diagnosis is designated as a medical act, and medical act must take place in medical institution. Using this web-based system, we can make primary diagnosis from anywhere in the world. But in accordance to the law, we kept the report as draft when made outside of the medical institution and it was later finished at the hospital.

Because we cannot access the hospital’s pathology reporting system directly from Tokyo, we cannot write the diagnosis directly. At least, this could help us because the pathology technicians can find simple mistakes in the report when they copy it.

## Conclusions

There are many ideas reported for the usage of VS and Telepathology [2-5]. This time, we focused on the web-based primary diagnosis. We constructed this system as a kind of emergency response for the absence of a pathologist, and it proved to be useful. There still many issues that remain, like the way to write diagnosis report to the system, clarification of where responsibility lies, and how to handle the large operation specimens or cytology slides, etc. In Japan, we have a guideline for intraoperative frozen section telepathology diagnosis written by the Japanese Research Society of Telepathology and Pathology Informatics [6]. We may require a guideline for web-based remote primary diagnosis using VS.

## Competing interests

The authors declare that they have no competing interest.

## Authors' contributions

IM did the most remote diagnosis. TO actually visit the hospital, look slides by microscope, and made many discussion with IM. YM participate to the VS telepathology and gave many suggestions. TI and HU are the pathology technicians of the hospital who prepare pathology slides, scan to VS, and copy the diagnosis to the system. TS and YO are experienced pathologists who gave suggestion to the design of the study, and helped to draft the manuscript.
